# Artificial intelligence and laboratory biomarkers in veterinary medicine: an update about machine learning applications

**DOI:** 10.1080/01652176.2026.2715555

**Published:** 2026-09-17

**Authors:** Fernando Tecles, Julian J. Arense-Gonzalo, Alberto Muñoz-Prieto, Asta Tvarijonaviciute, Silvia Martínez-Subiela, Edgar G. Manzanilla, José J. Cerón

**Affiliations:** a Salilab-UMU, Interdisciplinary Laboratory of Clinical Analysis, Veterinary School, Regional Campus of International Excellence ‘Campus Mare Nostrum’, University of Murcia, Murcia, Spain; b Multidisciplinary Institute of Biomarkers Research (Imubi), Murcia, Spain; c Pig Development Department, Moorepark Animal and Grassland Research Centre, Teagasc, Irish Agriculture and Food Development Authority, Cork, Ireland; d School of Veterinary Medicine, University College Dublin, Dublin, Ireland

**Keywords:** Artificial intelligence, biomarkers, bovine, companion animals, machine learning, swine, algorithm, review

## Abstract

The use of artificial intelligence has been a revolution in human medicine in the last years. Machine learning (ML), a subset of artificial intelligence, has allowed the use of a large amount of data to search for associations of clinical significance. Although the use of ML with laboratory biomarkers in veterinary medicine is limited in comparison with the human side, the number of studies published in this field is increasing in recent years. This review addresses some basic concepts and gives a general overview of the use of ML in the area of biomarkers in veterinary medicine. It is especially focused on two species of each companion (dogs and cats) and farm (bovine and porcine) animals, and provides information about the advances made in the last years, indicating how ML using laboratory data can contribute to the diagnosis and monitoring of selected diseases and, in case of farm animals, also to better control and improve their productive performance. In addition, possible new applications such as optimizing laboratory management, refining diagnostics or improving treatment monitoring based on the experience in humans are outlined. This narrative review can contribute to a better knowledge about the possible use of ML in laboratory biomarkers in veterinary and animal sciences for current and future applications.

## Introduction

Artificial intelligence (AI) can be described as a group of computational methods that learn patterns from data to support complex tasks such as prediction, classification, or decision-making. Compared with conventional statistical methods, which typically depend on predefined model frameworks and assumptions regarding data distributions, AI techniques are capable of learning complex, non-linear patterns directly from data while requiring fewer prior assumptions (Baştanlar and Özuysal 2014) and make algorithms (a set of mathematical instructions or rules) that explain those relations (Waljee and Higgins 2010). As a result, in certain contexts (particularly with complex datasets) AI models may outperform traditional statistical approaches in making predictions and outcomes (Nishi et al. 2019; Quesada et al. 2019; Macesic et al. 2020), although this is not always achieved (Gravesteijn et al. 2020). For example, in a review made about AI clinical prediction modelling some years ago, the authors did not find better performance over regression-based models (Christodoulou et al. 2019).

Machine learning (ML) is a subset of AI that learns from structured or engineered data, whereas Deep learning (DL) is a subset of ML that learns directly from data, often reducing manual feature engineering and making it especially useful for complex inputs such as images or signals (Neal et al. 2025). In medical research, ML is especially useful in situations where large amounts of data are available or/and data is heterogeneous. This data can be of a different nature, including physical examination, imaging, and/or laboratory results. Whereas the study of all this data by the classical way of clinical examination provides a limited amount of information and can only establish those relationships that the clinician's eye can discern, ML can integrate all this information to find subtle patterns that otherwise would be unnoticed (Kukar et al. 2021).

A biomarker can be defined as a measurable feature that can be used as an indicator of physiological or pathological processes, as well as responses to a therapeutic intervention (Califf 2018). From the point of view of the clinical pathology laboratory, a biomarker is a biological molecule found in blood, other body fluids, or tissues that is a sign of a normal or abnormal process, or of a condition or disease, and that can be measured in laboratory conditions (Puntmann 2009). In clinical practice, a large amount of laboratory biomarker data is usually obtained, which can be analyzed by ML to identify interrelationships that could be indicative of specific clinical situations.

ML systems have been implemented in human medicine for many years (Handelman et al. 2018) to improve disease diagnosis (Goldstein et al. 2017; Lidströmer et al. 2022) or to establish personalized and more accurate treatments (Vranas et al. 2017; Choi et al. 2020). Specifically, in laboratory medicine, they have been used to automate laboratory tasks (Rabbani et al. 2022) and aid healthcare professionals in test result interpretation and taking clinical decisions (Çubukçu et al. 2024). Despite being used in human medicine for several years, ML could be considered a novel discipline in veterinary medicine, in particular in laboratory analysis (veterinary clinical pathology) (Neal et al. 2025).

The aim of this narrative review is to: (1) provide general concepts of the different types of ML that can be used with laboratory data, and how the results can be evaluated; (2) indicate recent studies available in veterinary medicine in which ML is applied to biomarkers in organic fluids, in two species of companion (dogs and cats) and two species of production animals (cows and pigs), in order to provide an overview of its current applications in veterinary medicine; and (3) provide examples of studies in humans that could be a guide for future advances in the veterinary laboratory side.

## General concepts

**Figure 1.**
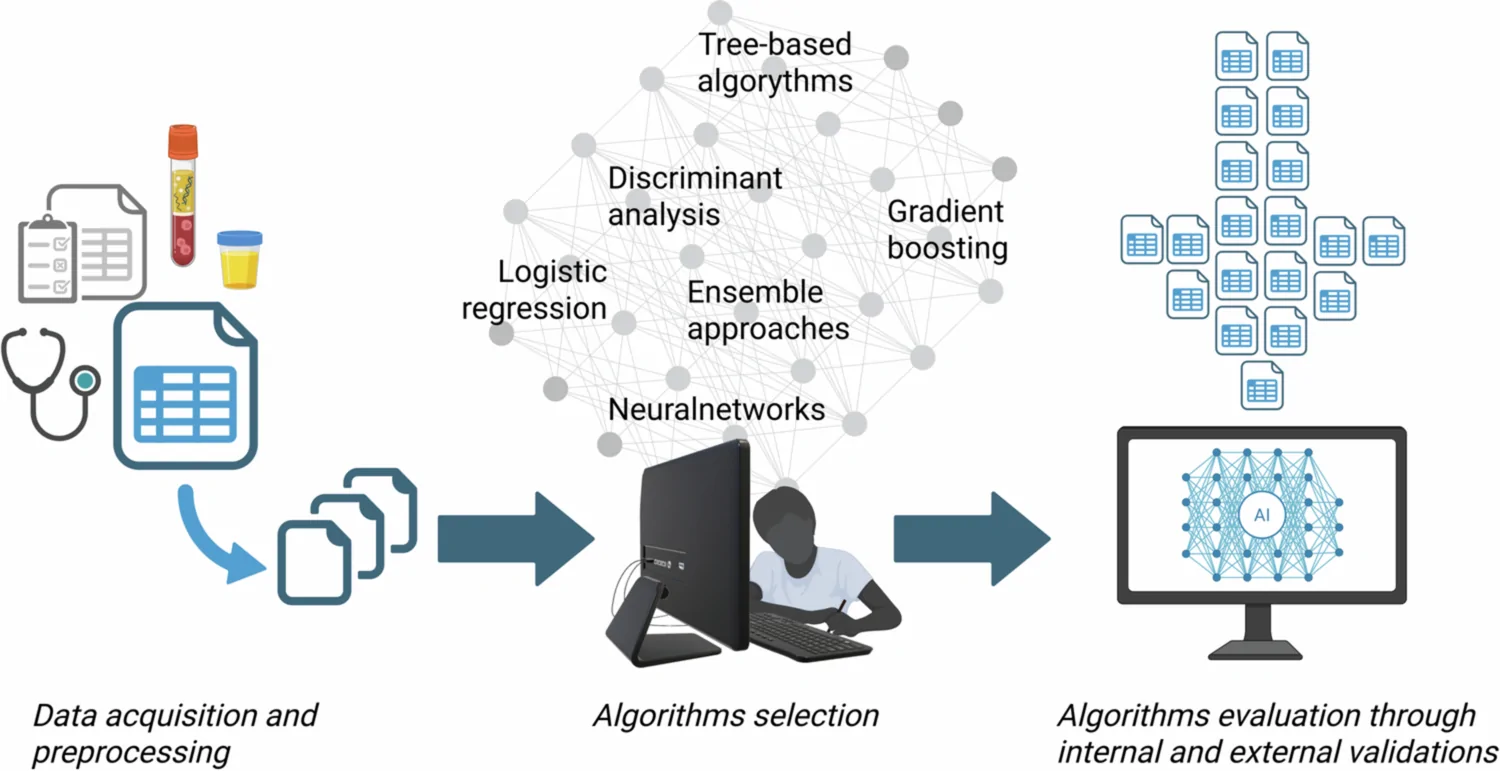
Steps for the development of ML models. Created with BioRender. Ceron, J. J. (2026) https://BioRender.com/pkghdeb.

Previous reviews about general ML concepts and their application in clinical analysis have been published where more detailed information can be found (Neal et al. 2025; Radin and Sprague 2025; Antunes and Kemiyondo 2026). To provide a general overview, that can specially be useful for people from the laboratory side to be introduced to the field of ML, the usual procedure for developing an ML model is as follows (Figure 1):

### Patient data acquisition and preprocessing

Data is classified as: (a) ‘predictors’, which are the different input variables that are included with the data. In laboratory medicine, the predictors are the different analytes that are included in a biochemical profile or cell blood count. (b) ‘The label’ or ‘target variable’, which is the outcome the model is trained to predict. In laboratory medicine can be, among others, the detection of a specific disease, clinical complications or the prediction of survival in a diseased animal (Luo et al. 2016).

As a first step in ML, the quality and structure of the input data must be ensured through a series of preprocessing steps. In these steps, some actions can be undertaken such as the integration of laboratory data with other clinical information, such as physical examination findings or imaging-derived variables, that can enhance both model performance and clinical applicability (Yan et al. 2023). In addition, data may need to be cleaned, that consists in the correction or removal of erroneous, inconsistent, or missing values; outlier detection; and the application of selection techniques to remove redundant or irrelevant variables. Also, in some contexts, variables should be transformed to a comparable numerical range, particularly when using certain ML algorithms listed in Table 1, such as k-nearest neighbors or support vector machines. Class imbalance, that means that one of the categories of the population to be studied is substantially underrepresented, should also be addressed at this stage; for this, there are strategies such as resampling techniques (e.g. oversampling, undersampling) that can avoid a model biased toward the majority class. Detailed information about how to handle data integration, cleaning, transformation and class imbalance can be found in previous reviews (Albahra et al. 2023; Niazy et al. 2025).

**Table 1.** Main machine learning algorithms and technical characteristics (Hofmann et al. 2008; Hastie et al. 2009; Fernández-Delgado et al. 2019; Kyriazos and Poga 2024; Al-Nafjan et al. 2025).

Algorithm	Learning type	Main family	Subfamily	Technical justification
Linear regression	Supervised	Linear/penalized regression	Parametric linear model	Interpretable baseline model for simple relationships and validation of individual biomarkers
Logistic regression (LASSO/Ridge)	Supervised	Linear/penalized regression	Penalized linear model	Enables feature selection in high-dimensional omics datasets
Decision tree	Supervised	Tree-based models	Classification and regression trees	Interpretable partitioning of feature space; captures simple non-linear interactions
Random forest	Supervised	Tree ensembles	Bagging of trees	Reduces variance and handles noisy high-dimensional data
Gradient boosting	Supervised	Ensemble	Additive boosting	Sequentially improves predictive performance on complex tabular data
Extreme boosting	Supervised	Ensemble	Regularized boosting	Optimized boosting with regularization; widely used in biomedical datasets
Support vector machine	Supervised	Kernel methods	Max-margin classifier	Effective in high-dimensional spaces; kernel trick captures non-linear relationships
K-nearest neighbors	Supervised	Instance-based methods	Lazy learning	Relies on distance metrics; performance degrades in high-dimensional settings
Naive Bayes	Supervised	Probabilistic generative models	Bayesian classifier	Efficient under high dimensionality with independence assumptions
Neural networks (MLP)	Supervised	Neural networks/deep learning	Feedforward	Flexible nonlinear modeling; requires larger datasets
Convolutional neural network	Supervised	Neural networks/deep learning	Convolutional networks	Suitable for structured data such as imaging or signals
Recurrent neural network	Supervised	Neural networks/deep learning	Recurrent networks	Captures temporal dependencies in longitudinal biomarker data
Principal component analysis clustering	Unsupervised	Dimensionality reduction/chemometrics	Linear projection	Reduces dimensionality and collinearity; common in omics analysis
K-Means	Unsupervised	Clustering	Centroid-based	Partitions samples into clusters; sensitive to initialization
Density-based spatial clustering of applications with noise	Unsupervised	Clustering	Density-based	Identifies clusters of arbitrary shape and handles noise

Supervised: the model is trained with labelled data; Unsupervised: the model is trained with unlabeled data.

### Establishment of the algorithms: training and validation

The most used algorithms in laboratory medicine for ML, arranged according to their family and their main characteristics, are summarized in Table 1. Some of them operate in a supervised manner, where the model is trained on labelled or previously classified data (e.g. healthy vs. diseased subjects or subjects with a specific disease or clinical condition), to predict specific outcomes. While others operate unsupervised, analyzing unlabeled data where the model is left to describe relationships in the data according to trends that it observes to discover previously unknown patterns. Algorithms such as decision trees and random forest are examples of supervised, whereas K-means is an example of an algorithm used in unsupervised data.

Algorithm selection should be driven by different factors such as sample size, input modality (e.g. unimodal including numerical results only vs. multimodal in which numerical data is combined with data of other nature such as images), the deployment context that is the real-world operational where the model will function for the final user (e.g. hospital environment vs. field conditions) and/or interpretability requirements (whether the model decision can be correctly interpreted) (Thomas and Rajabi 2021; Chen et al. 2023; Liu et al. 2024; Antunes and Kemiyondo 2026). For this reason, no single algorithm is expected to perform best across all different datasets. Therefore, a common approach consists in the use and comparison of various ML algorithms on the same data set to identify the one that yields the best results (Baysal Erez et al. 2025), as it can be seen in most of the studies included in this review (Tables 2 and 3).

**Table 2.** Reports about machine learning using laboratory data in dogs and cats between 2019 and 2025.

Species	Purpose and reference	Input modality and sample size	Task type	Algorithm/s tested	Type of validation	Selected algorithm	Best metrics	External validation
Dog	Identification of cardiomegaly in dogs with degenerative mitral valve disease (Wilshaw et al. 2021)	Unimodal/tabular: clinical observations, biochemistry and cardiac biomarker concentration from 1245 dogs	Classification	LR, SVM, RF, XGBoost	Training and validation sets	BLR	84% probability to identify cardiomegaly	No
	Visceral leishmaniasis diagnosis (Larios et al. 2021)	Unimodal/tabular: Fourier transform infrared spectroscopy of serum from 48 dogs	Classification	KNN, SVM, DA	Leave one out cross validation	KNN	85.42% accuracy classifying non-infected sera, and sera infected with *Leishmania infantum* and *Trypanosoma evansi*	No
	Hypoadrenocorticism diagnosis (Reagan et al. 2020)	Unimodal/tabular: CBC and serum chemistry from 1041 dogs	Classification	ABoost	Training and validation sets	NA	Overall AUC of 0.99	No
	Predict survival after canine parvovirus infection (Franzo et al. 2020)	Unimodal/tabular: anamnestic, physical examination, hematological and biochemical parameters from 137 dogs PCR positive to CPV	Classification	RF, LR	Training and validation sets	RF	Overall accuracy of 97.5%	No
	Cushing’s syndrome diagnosis (Schofield et al. 2021)	Unimodal/tabular: demographic data, clinical signs and laboratory data (ALP and ALT) from 1000 dogs (419 with Cushing and 581 suspect but non-cushing)	Classification	LASSO, RF, SVM, RBF	Training and validation sets	LASSO	Overall AUC of 0.85	No
	Leptospirosis infection diagnosis (Reagan et al. 2022)	Unimodal/tabular: CBC, serum chemistry and urianalysis data from 466 dogs (100 positive and 366 negative) the first day of hospitalization	Classification	SVM	Training and validation sets	NA	AUC at validation set 0.955	No
	Subtyping PA (Kaneko et al. 2021)	Unimodal/tabular: blood biochemistry and physiological data from 229 dogs with PA	Classification	LR, SVM, RF, DT	Training and validation sets; 10-fold cross validation	RF	85.1% overall accuracy	Yes
	*B. canis* infection diagnosis (Pijnacker et al. 2022)	Unimodal/tabular: CBC data from 100 positive and 6793 negative dogs	Classification	LR, DT, RF, XGBoost	Training and validation sets; 10-fold cross validation	All of them	AUC > 98% in all approaches	No
	*L. infantum* infection diagnosis (Coelho et al. 2023)	Unimodal/tabular: UV spectra of serum from 52 dogs (26 positive and 26 negative to *L. infantum*)	Classification	DA, SVM, KNN	Training and validation sets; 10-fold cross validation	SVM	Overall accuracy for correct classification: 75%	No
	Hypothyroidism diagnosis (Corsini et al. 2023)	Unimodal/tabular: clinical and clinicopathological parameters from 315 dogs (233 euthyroid and 82 hypothyroid)	Classification	CT, RF, GBM, SVM, LR, NB	10-fold cross validation	LR without thyroid hormones RF with thyroid hormones	87% accuracy when thyroid hormones are not included 97% accuracy when thyroid hormones are included	No
	Canine mammary tumor diagnosis (Enginler et al. 2024)	Unimodal/tabular: serum and tissue homogenates electrochemical data from 17 bitches with canine mammary tumors and 1 control bitch	Classification	RF, XGBoost, LightGBM, NN	Leave one out cross validation	RF	Overall accuracy 98%	No
	PPS diagnosis and classification (Farhoodimoghadam et al. 2024)	Unimodal/tabular: demographic, CBC and serum chemistry data from 652 PPS and 589 non-PPS (other diseases) dogs	Classification	XGboost	Training and validation sets	NA	97.6% AUC for PPS diagnosis 85.7% accuracy for PSS sub-classification	No
	Predicting outcome in CPV (Sanaei et al. 2025)	Unimodal/tabular: SIRS, deworming and vaccination frequency, and crying behavior from 156 dogs with CPV infection	Classification	LR, SVM, GPC, DT, RF, ABoost, NB, DA, GBM	Training and validation sets	NA	Approaches average accuracy 84% (ranging 83%–85%)	No
	Tumor diagnosis (Sharif et al. 2025)	Unimodal/tabular: combination of patient age, serum TK1 and serum CRP levels from 220 bearing tumors dogs and 67 healthy dogs	Classification	GBM, DT, RF, LR	Training and validation sets; 10-fold cross validation	LR	98% AUC for correctly classifying tumour-bearing dogs	No
Cat	Early detection of chronic kidney disease (Bradley et al. 2019)	Unimodal/tabular: creatinine, blood urea nitrogen, urine specific gravity and age from 106,251 cats with at least two visits	Classification	NN	Training and validation sets	ND	90.7% sens and 98.9% spec near diagnosis 63.0% sens and 99% spec 1 year before diagnosis	No
	Predicting survival in acute-on-chronic kidney disease (Renard et al. 2021)	Unimodal/tabular: clinical and analytical data from 42 cats	Classification	DT	No validation	ND	96% sensitivity and 53% specificity for predicting survival at 7 days of presentation	No
	Intestinal disease diagnosis (Basran et al. 2023)	Multimodal/tabular and imaging: abdominal ultrasound images, CBC, and blood serum analysis from 149 cats	Classification	SVM, KNN, RF, RF2, NN, NB	Training and validation sets; 10-fold cross validation	In general, no differences were seen in accuracy between ML approaches with the exception of NB that provided lower accuracy	Accuracy > 88%, being > 86% when using only CBC and serum biochemistry	No
	Early detection of CKD (Vanden Broecke et al. 2025)	Unimodal/tabular: metabolomic, serum biochemistry and clinical data from 87 healthy and 85 diseased cats	Classification	LASSO, LIMMA, RF, SVM	10-fold cross validation	SVM	92.9% AUC 6 months before traditional diagnosis	No
Dog/Cat	Survival after diaphragmatic herniorrhaphy (Kwananocha et al. 2025)	Unimodal/tabular: clinical and laboratory data from 126 cats and 63 dogs with diaphragmatic herniation	Classification	LR, RF, DT	10-fold cross validation	NA	All approaches found duration of herniation and BUN concentration as significant predictors of survival	No

Abbreviations: ABoost: adaptative boosting; ALP: alkaline phosphatase; ALT: alanine aminotransferase; AUC: area under the curve; BUN: blood urea nitrogen; CBC: complete blood cell count; CKD: chronic kidney disease; CPV: canine parvovirus; CT: classification tree; DA: discriminant analysis; DT: decision tree; GBM: gradient boosting machine; GBT: gradient boosted tree; GPC: Gaussian process classifier; KNN: K-nearest neighbors; LASSO: least absolute shrinkage and selection operator; LightGBM: light gradient boosting machine; LIMMA: linear model for microarray data; LR: logistic regression; ML: machine Learning; MLP: multilayer perceptron; NA: not applicable; NB: Naïve Bayes algorithm; NN: neural network; PA: primary aldosteronism; PPS: portosystemic shunt; RBF: radial basis function; RF: random forest; RF2: RUBoost random forest search algorithm; SVM: support vector machine; XGBoost: extreme boosting algorithm.

**Table 3.** Reports about machine learning using laboratory data in cows and pigs between 2019 and 2025.

Species	Purpose and reference	Input modality and sample size	Task type	Algorithm/s tested	Type of validation	Selected algorithm	Best metrics	External validation
Cow	Differentiate between high body condition (HBCS) and normal body condition (NBCS) cows (Ghaffari et al. 2019)	Unimodal/tabular: serum biochemistry and metabolomics from 38 cows (19 HBCS and 19 NBCS). Samples were collected sequentially from 49 before to 84 after calving	Classification	RF	Leave one out cross validation	NA	68.4% accuracy on day 49 before calving 60.5% accuracy on day 21 after calving	No
	Prediction of dry matter intake (DMI) and residual feed intake (RFI) (Martin et al. 2021)	Unimodal/tabular: combinations of descriptive, performance, sensor-derived behavioral, and blood metabolite data from 124 Holstein cows	Regression	LR, PLS, NN, SEB	Training and validation sets; 5-fold cross validation	LR for DMI prediction any approach useful for RFI prediction	R^2^ > 0.78	No
	Prediction of metabolic status (Xu et al. 2019)	Unimodal/tabular: milk composition and performance data from 295 cows	Classification	DT, NB, BY, KNN, SVM, ANN, RF, BA	10-fold cross validation	RF SVM	FR: highest sensitivity (67.8%–82.9%) SVM: highest specificity (80.9%–93.7%)	No
	Udder health status prediction (Bobbo et al. 2021)	Unimodal/tabular: 18,442 records including production and data from milk analysis	Classification	LDA, GLM, NB, CRT, KNN, SVM, RF, NN	Training and validation sets; 10-fold cross validation	NN	AUC > 0.82	No
	Pregnancy prediction after artificial insemination (Ferraz et al. 2024)	Unimodal/tabular: genetic expression, blood perfusion and plasma levels of progesterone on 21 days after insemination of 140 cows and 32 heifers	Classification	SVM, RF, MAR, NN	Training and validation sets; 10-fold cross validation	MAR	Highest balanced accuracy (72%)	No
	Neonatal calf diarrhea diagnosis (Ceran and Gurbanov 2025)	Unimodal/tabular: serum infrared spectra from 30 calves (10 healthy, 10 with diarrhea and 10 recovered)	Classification	SVM, LDA	Training and validation sets; 30-fold cross validation	LDA	Perfect classification of the three groups (100% accuracy)	No
	Prediction of serum metabolites levels (Giannuzzi et al. 2023)	Unimodal/tabular: milk infrared spectra from 2741 cows	Regression	PLS, RF, XGboost, EN, GBM, NN	5-fold cross validation	EN and combinations of different ML approaches	Better R^2^ obtained for most of the analytical parameters	No
	Prediction of hematochemical parameters levels (Giannuzzi et al. 2022)	Unimodal/tabular: milk infrared spectra from 385 cows	Regression	NN, RF, GBM, EN	10-fold cross validation; leave one out cross validation	NN and combinations of the different ML approaches	They predicted correctly the highest number of analytes	No
	Predict dyscalcemia in early-lactation (Seminara et al. 2025)	Unimodal/tabular: milk productivity and metabolites data from 542 cows (433 eucalcemic and 109 dyscalcemic) at 4 DIM	Classification	PLS, LASSO, RR, SVM, RF	50-fold cross validation	RF	Overall accuracy 80%	No
	*Brucella abortus* detection (de Rezende et al. 2023b)	Unimodal/tabular: spectroscopy data of 106 bovine serum samples (53 positive and 53 negative to *B. abortus*)	Classification	DA, SVM, KNN	Training and validation sets; leave one out cross validation	KNN	Overall accuracy 95.9%	No
	Early identification of pregnant cows after artificial insemination (Reis et al. 2024)	Unimodal/tabular: near-infrared serum spectra from 60 cows collected just before artificial insemination	Classification	SVM	Leave one out cross validation	NA	100% accuracy for predicting pregnant cows after artificial insemination	No
	Subclinical ketosis diagnosis (Satoła and Bauer 2021)	Unimodal/tabular: combination of productivity data and milk composition analysis from 833 cows	Classification and regression	LR, EN, SVM, DT, ABoost, RF, RR, XGboost, GBM, KN	Training and validation sets; 10-fold cross validation	SVM used as classification modeling	It provided the best sensitivity (57%) and specificity (72%)	No
	Lameness diagnosis (Džermeikaitė et al. 2025)	Unimodal/tabular: behavioral, physiological, biochemical, and milk composition parameters collected from 272 dairy cows (110 lameness, 162 healthy controls) at early lactation	Classification	RF, NN, SVM, KN, LR	Monte Carlo cross validation	RF	Overall accuracy 97.04%	No
	Metabolic and udder health disorders diagnosis (Girdauskaitė et al. 2025)	Unimodal/tabular: behavioral and milk composition data from 164 cows (35 healthy, 75 subclinical ketosis, 20 subclinical mastitis and 34 clinical mastitis)	Classification	RF, GBM, LR, DT, MP	Training and validation sets; k-fold cross validation	RF	Overall accuracy 85.7%	No
	Hyperketonemia diagnosis (Walleser et al. 2023)	Unimodal/tabular: milk infrared spectroscopy data from 5747 cows (20,060 samples in total)	Classification	LDA, EN, NN, Deep learning models, Combinations of the above	Training and validation sets; 5-fold cross validation	NA	Balanced accuracy ranging 71.2%–78.1% between the different approaches	No
Pig	Predicting four fertility phenotypes: gestation length (GL), the total number of piglets born (TNB) per litter, the total number of stillborn piglets (TNS) per litter, and the total number of piglets born alive (TNA) per litter (Kamphuis et al. 2020)	Unimodal/tabular: data from 9418 ejaculates (634 boars), including fertility phenotype records, computer assisted semen analysis information, boar ejaculate information, breeding value estimations and weather information	Classification and regression	GBM	Training, validation and test sets	NA	Maximum AUC of 0.989 for the training set, dropped to 0.605 for validation set and 0.624 for an independent data set	No
	Diagnosis of different diseases (Llamas-Amor et al. 2025)	Unimodal/tabular: results of different salivary analytes from 344 pigs with different conditions (97 healthy; 118 *S. suis* infection; 77 ETEC; and 52 PRRS)	Classification	BLR, DT	Training and validation sets	BLR	81.8% and 89.7% overall accuracies for predicting ETEC and PRRS, respectively	No
	Perinatal hypoxia diagnosis (Lafuente et al. 2022)	Unimodal/tabular: physiological and arterial blood parameters from 22 newborn piglets before and after ischemia induction by carotid clamping (49 samples at basal conditions and 49 samples after induction)	Classification	PLS, RF, SVM, ABoost, GBM, XGboost, Combinations of above	5-fold cross validation	NA	Overall accuracies between 93.0%–94.5% with all approaches, although sensitivity showed improvements with combinations	No

Abbreviations: ABoost: adaptative boosting; ANN: artificial neural network; BA: bootstrap aggregation; BLR: binary logistic regression; BN: Bayesian network; DA: discriminant analysis; DT: decision tree; EFS: ensemble feature selection; EN: elastic net; ETEC: enterotoxigenic *Escherichia coli*; GBM: gradient boosting machine; GTB: gradient tree boosting; IGR: information gain ranking; KNN: K-nearest neighbors; LASSO: least absolute shrinkage and selection operator; LDA: linear discriminant analysis; LR: linear regression; MAR: multivariate adaptative regression; ML: machine Learning; MP: multilayer perceptron; NA: not applicable; NB: Naïve Bayes; NN: neural networks; PCA: principal component analysis clustering; PLS: partial least squares; PRRS: porcine reproductive and respiratory syndrome; RF: random forest; ROC: receiver operation characteristics assay; RR: ridge regression; SVM: support vector machine; XGboost: extreme boosting algorithm.

To internally validate an ML algorithm, a hold-out validation can be done, in which a first step called the ‘training’ of the algorithm using a training dataset to fit the parameters of an ML model is performed. Then, there is a second step called the ‘testing’ of the algorithm, which uses a testing dataset (independent of the training dataset) to assess the predictive performance of the model (Reel et al. 2021). As an example, in some studies 70%–80% of data have been used for training and the remaining 20%–30% for testing (Walleser et al. 2023; Farhoodimoghadam et al. 2024), although alternative ratios can be used depending on the data set and there are reports about the ideal split ratios (Tan et al. 2021; Joseph 2022). Another procedure is to perform a K-fold cross-validation, in which the training dataset is randomly split into K equal-sized subsets (called folds), and the model is trained and tested K times (K-1 folds are combined to train the model and the remaining fold is held back as the validation set). This process is repeated until every single fold has served as the validation set once (Bradshaw et al. 2023).

At training and testing steps, the metrics used to evaluate the performance of the ML algorithm are common clinical parameters such as accuracy, sensitivity, specificity, precision, receiver operating characteristic (ROC) curves, or area under the curve (AUC). In some reports, sensitivity is also called recall and it is used to calculate the F1-score which is the harmonic mean of precision and recall. A more complete evaluation should include confidence intervals, uncertainty of all key metrics, prevalence effects and explicit decision thresholds linked to the clinical use. Also, calibration should be performed after testing the model to ensure the agreement between predicted probabilities and actual observed frequencies. More detailed information about these parameters can be found in previous reports (Collins et al. 2014; Cabitza et al. 2021; de Hond et al. 2023).

Ideally, an ‘external validation’ should be made in order to evaluate whether the model demonstrates sufficient robustness and generalizability to support their implementation in routine clinical veterinary practice. This should cover whether the model generalizes to new cohorts of animals usually obtained from a different institution, geographical region, or study population. In this step, from the laboratory point of view, the variations due to the possible use of different assays for analyte measurements should be taken into consideration, and ideally the assays used to generate data for the algorithm in other laboratories should give similar results in interlaboratory quality control material. There are many authors who indicate that external validation is indispensable since it tests transportability in independent data (Collins et al. 2014; Cabitza et al. 2021; de Hond et al. 2023). However, this procedure is not commonly performed, as it can be seen in this review, given that only one of the included articles conducted a strict external validation.

## Articles selection

**Figure 2.**
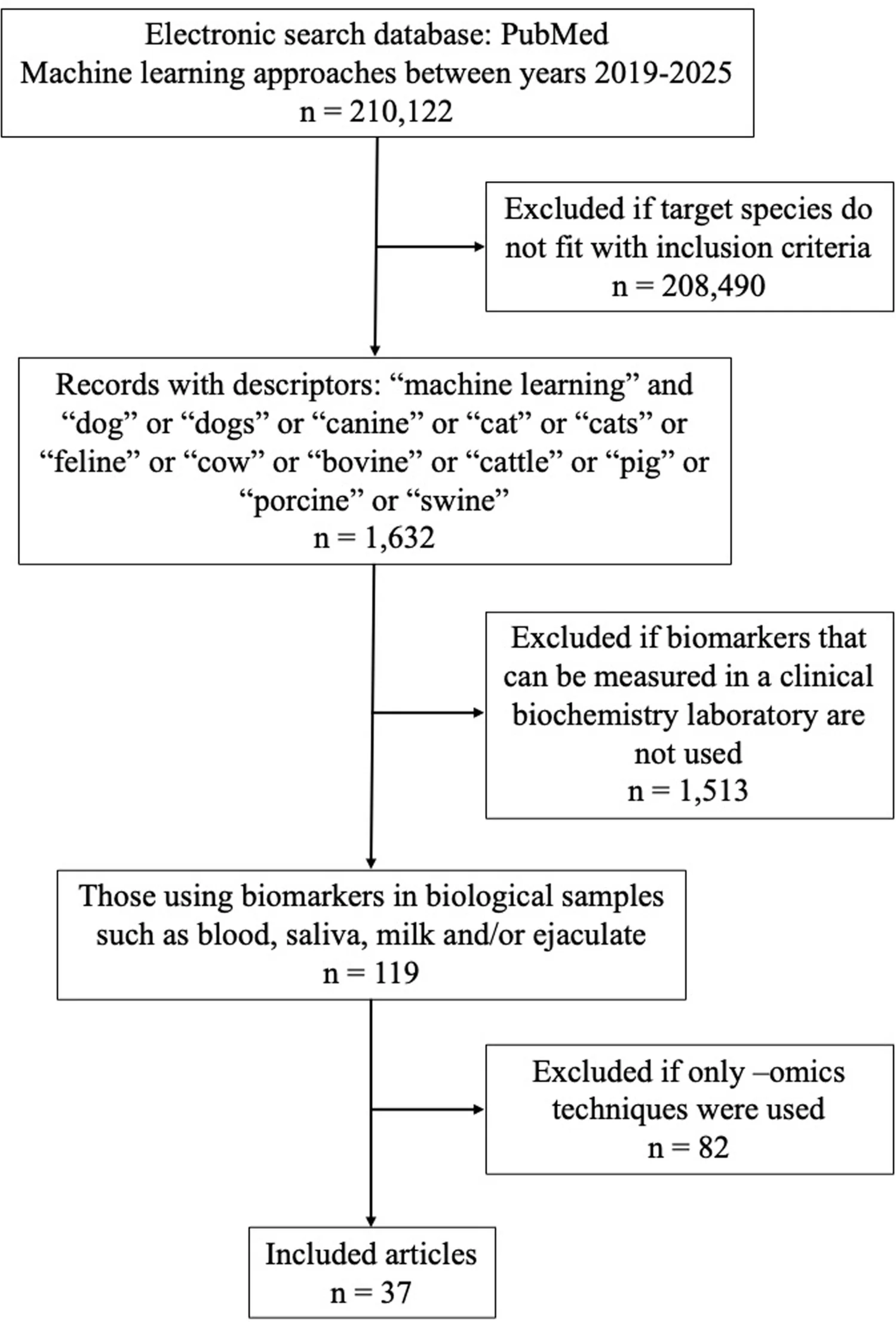
Inclusion and exclusion criteria used for article selection.

The search was carried out using the PubMed platform. The inclusion criteria included articles published between the years 2019 and 2025 (both included) about machine learning approaches with the following characteristics: (a) in canine, feline, bovine and swine species; (b) models were developed including biomarkers that can be measured in a clinical biochemistry laboratory in different biological fluids (blood, saliva, milk and/or ejaculates) as predictors, alone or together with other inputs such as clinical observations, behavioral data, imaging or other clinical data. Exclusion criteria included: (a) species different from those previously specified in the inclusion criteria; (b) no analytical biomarkers that can be measured in a clinical biochemistry laboratory were used; (c) or if only ‘omics’ techniques were used. The studies were selected by direct reading of them, avoiding duplicities if needed. The procedure is summarized in Figure 2.

It is important to highlight that this review is descriptive and not analytical, so it is limited to show the studies published in the field of ML indicating sample size, tested algorithms and performance, as well as the kind of validation performed, but without assessing their suitability and risk of bias.

## Machine learning in biomarkers in dogs and cats

The search in PubMed between years 2019–2025 with the terms ‘machine learning’ and ‘dog’, ‘dogs’, ‘canine’ provided 274 entries. The terms ‘machine learning’ and ‘cat’, ‘cats’ or ‘feline’ provided 138. When the term ‘biomarkers’ was included, only 33 entries for dogs and 9 entries for cats were found between 2019 and 2025. The articles were reviewed to select only those using data obtained from biological samples, and those exclusively using -omics or DNA/RNA assays were excluded. Table 2 summarizes those studies (14 in dogs, 4 in cats, and 1 in both species), indicating the different types of ML algorithms used and, in the case of the use of various types, the performance data of the best one. Some examples of these studies are discussed in more detail in the following paragraphs.

To date, the main application of ML in biomarkers in companion animal medicine has been for diagnostic purposes. As an example, canine hypoadrenocorticism is a disease with a challenging diagnosis because the clinical signs resemble other illnesses such as kidney, gastrointestinal disorders or hepatic insufficiency (Klein and Peterson 2010; Zeugswetter and Schwendenwein 2014). This implies a high risk because this disease could be fatal if not diagnosed on time. Adrenocorticotrophic hormone (ACTH) stimulation test is required for definitive diagnosis (Feldman and Nelson 2004), but it can have limitations of high cost and, in some cases, lack of availability of synthetic ACTH. Thus, in an effort to provide new approaches for the diagnosis of the disease, ML identified a panel integrated by routine CBC and serum biochemistry analytes that provided 96% sensitivity and 97% specificity for hypoadrenocorticism diagnosis (Reagan et al. 2020). This algorithm was further applied in a cohort of 1025 dogs, achieving 100% specificity and 99.3% sensitivity for hypoadrenocorticism diagnosis (Reagan et al. 2022).

ML has also been applied for diagnosis of Cushing's syndrome, an endocrine disease that occurs due to an excessive secretion of cortisol (Feldman and Nelson 2004). In this case, several tests exist for its diagnosis, such as the ACTH stimulation test and the low-dose dexamethasone suppression test (LDDST). Those tests, however, have a variable and sometimes non-optimal sensitivity and specificity depending on the different studies, with ranges of 57%–83% and 59%–95%, respectively, for the ACTH stimulation test (Kaplan et al. 1995; Van Liew et al. 1997; Monroe et al. 2012; O'Neill et al. 2016; Nivy et al. 2018); and 85%–97% and 70%–73%, respectively, for the LDDST (Rijnberk et al. 1988; Van Liew et al. 1997; Bennaim et al. 2018). The ML developed included several variables such as breed, sex, neuter status, date of birth, body weight, clinical signs, and routine laboratory measurements such as urine specific gravity, urine protein to creatinine ratio, and alkaline phosphatase (ALP) and aspartate aminotransferase (ALT). This model showed 71% sensitivity and 82% specificity for the diagnosis of Cushing’s disease in dogs and it can contribute to a better triage of the patients to perform more specific tests (Schofield et al. 2021).

ML has also been used in companion animals to identify biomarkers that can predict survival in specific situations of disease. For this purpose, an algorithm has been developed to predict survival in cats with acute-on-chronic kidney disease (ACKD). This approach used several data including age, body weight, clinical score, as well as biomarkers (serum creatinine) and ultrasonographic data. The algorithm predicted survival with sensitivity over 70% and specificity over 50%, being serum creatinine the most important variable (Renard et al. 2021).

## ML in biomarkers in cows and pigs

The search in Pubmed between years 2019–2025 with the terms ‘machine learning’ and ‘cow’, ‘bovine’ or ‘cattle’ provided 680 entries; being 540 with the terms ‘machine learning’ and ‘pigs’ or ‘porcine’. Most of them included behavioral and productivity data. These data were collected manually by producers or veterinarians or automatically using sensors-robots, within the concept of precision livestock farming (PLF) (Piñeiro et al. 2019).

When the search was limited to those that included the term ‘biomarker’, 40 results were found for cows, and 37 results in the case of pigs. Then, when studies that only used -omics and DNA/RNA techniques were discarded, the number of studies was further reduced to 15 in cows and 3 in pigs (Table 3).

Two aspects of these studies are of particular interest:

- Other biological fluids apart from blood or serum have been used, such as milk in the case of bovine species. Milk analytical data can be used to detect mastitis, a severe disease characterized by an inflammatory condition affecting the mammary gland (Gelasakis et al. 2015). An algorithm has been created combining productive, environmental and milk analytical data to classify healthy and mastitis mammary glands, with an AUC over 82% (Bobbo et al. 2021). Another recent research developed predictive models using salivary analytes in pigs for detecting some specific diseases. The models combined different analytes and showed overall accuracies of over 78% and 87% for the detection of pigs affected by enterotoxigenic *Escherichia coli* and porcine reproductive and respiratory syndrome virus (PRRSv), respectively (Llamas-Amor et al. 2025). This research opens a new way for the use of saliva laboratory data in pigs, a species in which blood sampling is tricky.
- In addition to their clinical use in diseases, there are other applications such as to enhance productivity by detecting individuals with higher feed conversion, pregnancy prediction after artificial insemination and determination of the time of calving.

## Some issues and possible solutions

Several issues were identified across the reviewed studies. On the one hand, possibly there is a relatively limited sample size used in some papers. Additionally, cases of group imbalance (groups with different sample sizes) appeared. These issues can lead to model overfitting, which occurs when the model learns the training data memorizing its specific details, noise, and random fluctuations rather than capturing the underlying general patterns. As a result, the model achieves high performance on the training set but it fails to generalize and perform adequately in the validation one (Steyerberg et al. 2003).

Although ideally the studies should be designed to avoid these issues, in some situations they can be unavoidable, such as in real situations of non-frequent diseases or budget and/or resources limitations. In these cases, some techniques can be implemented to minimize overfitting as seen in reports of this review. For example, internal validations such as hold-out or cross validations have been used (only one article was found in which no validation was performed), which allows training the model minimizing overfitting. In other instances, authors used SMOTE (Synthetic Minority Over-sampling Technique) methods, which rely on mathematical procedures that duplicate existing records in the minority class to create completely new synthetic observations, aiming to train the algorithm on balanced populations to prevent overfitting toward the majority class (Chawla et al. 2002). In addition, weighted or normalized metrics such as F1-Score are also used since better evaluates performance when unbalanced data are used (Jeni et al. 2013). Furthermore, algorithms such as RF can reduce such overfitting and perform data rebalancing if any category is over- or underrepresented relative to the rest (Maw et al. 2022).

Another issue is that, in some cases, only a single algorithm was trained (8/37 articles of this report) and no comparisons were made with classical models such as linear or logistic regressions. This does not allow the evaluation of whether the use of ML-based algorithms yields a clear benefit over traditional mathematical models. Finally, only a single study was found in which a strict external validation of the model was performed using external datasets gathered by institutions other than the study's authoring institution, thereby allowing the generalizability of the obtained results to new cohorts.

## What comes next? Future machine learning applications in veterinary medicine based on human advances

There are various recent reviews available on applications of ML in biomarkers in humans. They outline the impact in diagnostic and patient care (Xie et al. 2024; Ashraf et al. 2025), clinical research and development (Mohapatra et al. 2025; Ko et al. 2025) or biomarker discovery (Javaid et al. 2025). In addition, guidelines for the use and application of ML-based biomarkers in some specific areas such as oncology have been recently published (Aldea et al. 2025).

Particular advances that can be obtained from the human side are:

### Advances in hospital organization

ML could help in hospital workload organization by predicting when dependencies and medical equipment bear the greatest workload to optimize their use, and also perform administrative tasks aimed at organizing the work of healthcare personnel or managing patient data (Hennrich et al. 2024). Work in the clinical laboratory could also benefit from the application of ML, not only in the management and interpretation of results, but also in the automation of procedures or in the improvement of quality controls (Xie et al. 2024).

**Figure 3.**
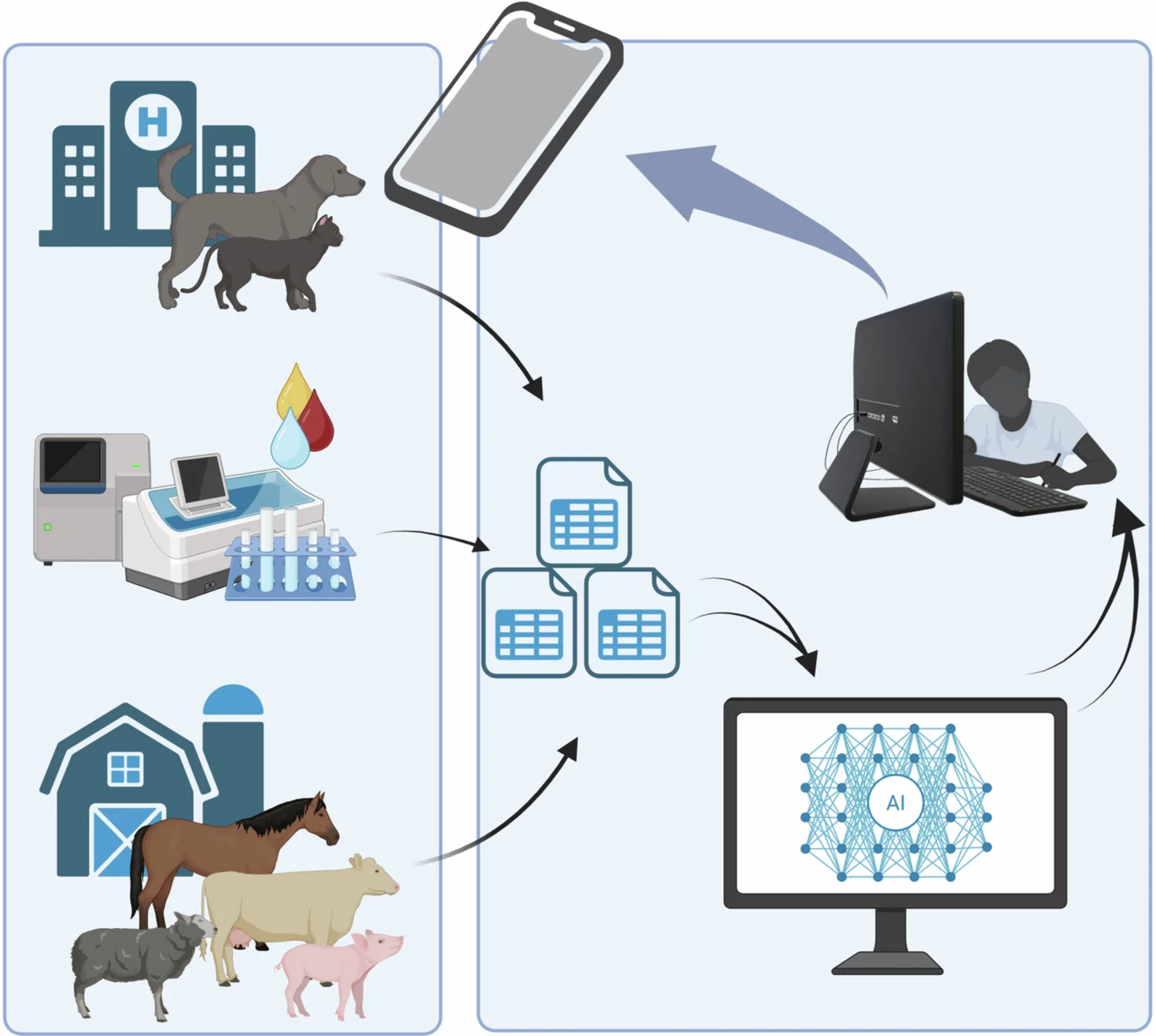
An example of how Machine Learning can be combined with mobile devices in companion and farm animals. The biomarker results that can be obtained in the laboratory or directly by the owners or farmers on site can be automatically analyzed by an Artificial Intelligence, interpreted and sent to users after being analyzed and verified by the clinician. Created with BioRender. Ceron, J. J. (2026) https://BioRender.com/pkghdeb.

In data management, ML can be combined with application programs (apps) on mobile devices in order to let the final users, in this case veterinary practitioners, get their results (Figure 3).

### Personalized medicine

Human hospitals can accumulate plenty of multidimensional data from patients, including genetic, proteomic, clinical, biochemical, temporal, demographic, and therapeutic (Handelman et al. 2018). ML can use all this data for several purposes, such as personalizing medicine (Holzinger 2014), recognizing the microvariables within each patient that can make the course of the disease different from others. This individualization could be at different levels: diagnosis (Dilsizian and Siegel 2014), prognosis and treatment (Cruz and Wishart 2006; Menden et al. 2013).

### Increasing achievement of accurate and early diagnosis

ML applications in human medicine increase the possibility of accurate disease diagnosis by sub-classifying subtypes within a specific disease. This is the case of primary aldosteronism (PA), which can be subdivided into unilateral or bilateral subtypes, which is an important fact because the unilateral subtype has an increased risk of cardiometabolic disease. A supervised ML classifier provided an accuracy of 95.7% and an AUC of 0.990 for predicting subtype diagnosis of PA, in which blood concentrations of sodium, potassium and aldosterone were identified as the most important predictors of the model (Kaneko et al. 2021).

Regarding early diagnosis, ML can be used to assess in advance the possible risk of suffering a specific situation or complication of a current disease. For example, a real-time risk for pediatric acute kidney insufficiency (AKI), as well as its severity, can be predicted by an algorithm that uses 15 different predictors including vital constants, laboratory data or medications, among others. The model was successful in predicting moderate to severe AKI prior to detection by conventional criteria with a median lead-time of 30 h with an AUC of 0.89 (Dong et al. 2021). ML can also predict the appearance of pneumonia after stroke (Li et al. 2020), kidney transplantation (Luo et al. 2020), and liver transplantation (Chen et al. 2021).

### Advanced patients follow up

The use of ML for treatment monitoring has been widely studied in human patients with cancer (Verma et al. 2024). In addition, ML including serum biomarkers has been used to predict the probability of recovery in Covid-19 (Tian et al. 2020).

### Solving challenges

Veterinary medicine can learn from the current challenges in AI applications in humans, such as the need of AI education and collaboration with specialists in this field to facilitate the integration of AI into diagnostic practices, the possible ethical questions related to privacy protection or the use of standardized formats in data repositories (Xie et al. 2024).

## Conclusions

The use of algorithms generated through ML techniques has experienced a major increase in recent years in human medicine, and in veterinary it can provide useful tools for the application of laboratory biomarkers in companion and farm animal species. ML can help in diagnosis and treatment monitoring under the concept of personalized medicine, and additionally in farm animals to detect and select most convenient productive traits. In addition, it can be used by the laboratories to optimize their resources, management and function. It is expected that the contribution of AI will continue to grow in the coming years, representing a very useful tool to allow a better and more accurate control of animal health and welfare.
